# Prevalence and incidence rates of laboratory-confirmed hepatitis B infection in South Africa, 2015 to 2019

**DOI:** 10.1186/s12889-021-12391-3

**Published:** 2022-01-06

**Authors:** Shelina Moonsamy, Melinda Suchard, Pavitra Pillay, Nishi Prabdial-Sing

**Affiliations:** 1 Centre for Vaccines and Immunology, National Institute for Communicable Diseases, Division of the National Health Laboratory Service, Johannesburg, South Africa; 2 Department of Biomedical and Clinical Technology, Faculty of Health Sciences, Durban University of Technology, Durban, South Africa; 3 Department of Chemical Pathology, School of Pathology, Faculty of Health Sciences, University of the Witwatersrand, Johannesburg, South Africa; 4 Department of Medical Virology, School of Pathology, Faculty of Health Sciences, University of the Witwatersrand, Johannesburg, South Africa

**Keywords:** Hepatitis B, Prevalence, Incidence, Acute, HBsAg, Anti-HBc IgM, Test positivity rates

## Abstract

**Background:**

Hepatitis B virus (HBV), a global public health threat, is targeted for elimination by 2030. As national HBV prevalence and incidence is lacking for South Africa, our study aimed to provide such data in the public health sector.

**Methods:**

We analysed laboratory-confirmed HBV data from 2015 to 2019 to determine annual prevalence and incidence rates of HBV infection per 100,000 population, HBsAg and anti-HBc IgM test positivity rates, and HBsAg and anti-HBc IgM testing rates per 100,000 population. Time trend and statistical analyses were performed on HBsAg and anti-HBc IgM test positivity rates.

**Results:**

The national prevalence rate of HBV infection per 100,000 population increased from 56.14 in 2015 to 67.76 in 2019. Over the five years, the prevalence rate was higher in males than females, highest amongst individuals 25 to 49 years old and highest in Gauteng province. The HBsAg test positivity rate dropped from 9.77% in 2015 to 8.09% in 2019. Over the five years, the HBsAg test positivity rate was higher in males than females, amongst individuals 25 to 49 years old and amongst individuals of Limpopo province. Amongst HBsAg positive children under 5 years old, the majority (65.7%) were less than a year old. HBsAg testing rates per 100,000 population were higher in females under 45 years of age and in males 45 years and above. The national incidence rate of acute HBV infection per 100,000 population dropped from 3.17 in 2015 to 1.69 in 2019. Over the five-year period, incidence rates were similar between males and females, highest amongst individuals 20 to 39 years old and highest in Mpumalanga province. Amongst individuals 20 to 24 years old, there was a substantial decline in the incidence and anti-HBc IgM test positivity rates over time. Anti-HBc IgM testing rates per 100,000 population were higher in females under 40 years of age and in males 40 years and above.

**Conclusion:**

Critical to hepatitis B elimination is strengthened infant vaccination coverage and interruption of vertical transmission. Transmission of HBV infection in adults may be reduced through heightened awareness of transmission routes and prevention measures.

**Supplementary Information:**

The online version contains supplementary material available at 10.1186/s12889-021-12391-3.

## Background

Hepatitis B, a global public health threat, is a potentially life-threatening viral infection of the liver caused by the hepatitis B virus (HBV) [1]. Transmission of HBV infection is through contact with blood or other body fluids of an infected person, mainly via vertical transmission from mother to child, sexual contact and the sharing of equipment between intravenous drug users [2]. In 2015, the World Health Organisation (WHO) estimated that 257 million people were living with chronic HBV infection, with the greatest burden in the African and Western Pacific Regions [3]. Chronic HBV infection has no known cure, with a 15-40% lifetime risk of cirrhosis, liver failure or hepatocellular carcinoma [4]. Globally, in 2013, there were more deaths due to viral hepatitis (1.4 million) than HIV infection (1.3 million) [5, 6]. More recent estimates from 2017 show that HBV accounted for 24% of cirrhosis-related deaths in females and 32% of cirrhosis-related deaths in males globally, and 20% of cirrhosis-related deaths in females and 23% of cirrhosis-related deaths in males in Southern sub-Saharan Africa [7]. In 2016, the WHO adopted a resolution to eliminate HBV infection by 2030, with the aim of reducing new chronic infections by 90% and reducing HBV related deaths by 65% [8]. South Africa has embraced this resolution, with subsequent development and approval of the national guidelines for the management of viral hepatitis [9].

A diagnosis of HBV infection requires laboratory confirmation by detection of HBV surface antigen (HBsAg), a marker of active HBV infection [10, 11]. The persistence of HBsAg for six months or more is indicative of a chronic infection [12–14]. Acute HBV infection occurs within the first six months following exposure to the virus and is defined by the presence of high levels of IgM antibody to the core antigen (anti-HBc IgM). Low-level anti-HBc IgM antibodies are usually associated with reactivation of HBV infection or flares amongst chronic carriers [15, 16].

The HBV vaccine is the backbone of prevention of HBV infection. South Africa introduced the vaccine into the expanded programme on immunisation (EPI) schedule in April 1995, administered as a monovalent dose at 6, 10 and 14 weeks of age (HepB3) [17]. Since December 2015, the HBV vaccine has been administered as a component of the hexavalent vaccine (DTaP-IPV-HIB-HepB), with an additional booster dose administered at 18 months of age [18, 19]. HepB3 reportedly induces protective antibody levels in approximately 95% of individuals [20]. It was further demonstrated that 51% of individuals who responded to the primary vaccination series had protective antibody levels 30 years later [21].

HepB3 coverage in South Africa, as per the District Health Information System (DHIS) and published by the Health Systems Trust of South Africa, averaged 76.6% for the period 2000 to 2018; with the lowest in 2001 at 67.0% and highest in 2006 at 85.5% [22–26]. The WHO-UNICEF estimates for South Africa for the period 2000 to 2018 averaged 76.1%, with the lowest in 2003 and 2010 at 71.0% and highest in 2014 and 2015 at 85.0% [27].

The WHO recommends a birth dose of the HBV vaccine (HepB_BD) to combat vertical transmission of HBV infection [28, 29]. Globally, by 2015, HepB_BD had significantly reduced the number of new chronic infections [8]. Although planned, South Africa is yet to implement HepB_BD, as transmission under five years of age was largely horizontal prior to vaccine introduction [17, 30]. Infants infected from their mothers have the highest risk of developing chronic HBV disease (70 to 90%). The risk decreases to between 20 to 60% in individuals who acquire infection between the ages of 1 and 6 years, 5 to 10% in individuals who acquire infection between the ages of 6 and 20 years, and 1 to 5% in individuals who acquire infection above the age of 20 years [31, 32]. On the contrary, the risk of developing symptoms in the acute phase of HBV infection increases with age. The risk of symptomatic HBV is around 1% in infants infected from their mothers, 10% in children 1 to 5 years of age and 30% in individuals over 5 years of age [12]. Antenatal HBV screening, recommended but not routinely performed in South Africa, would help assess and minimise the risk of vertical transmission through appropriate interventions [9].

Prior to vaccine introduction, hepatitis B was highly endemic in South Africa [19]. Following vaccine introduction, several sentinel studies reported HBsAg prevalence ranging from 2.9% in HIV-uninfected pregnant women in the Western Cape province in 2008 to 20.0% in HIV-infected individuals enrolled in workplace antiretroviral treatment programmes in 2002 and in HIV-infected individuals from North Eastern South Africa in 2008 [10, 33–37]. In children under 5 years of age, the overall HBsAg prevalence declined from 12.8% prior to vaccine introduction to 3.0% in 2009 [17, 28, 37]. In a study to determine HBV sero-prevalence in a convenience sample of children under 15 years of age, HBsAg prevalence was reported as 0.4% [19]. Data generated from some of these South African sentinel prevalence studies were used to develop a 5-year national hepatitis action plan using an investment case approach [38]. With the 2030 hepatitis B elimination goal fast approaching, our study aims to provide data on laboratory-diagnosed HBV infection in the public health sector at a national level.

## Methods

### Study population and data source

This was a retrospective quantitative cross-sectional study on routine HBV laboratory data obtained from the National Health Laboratory Service (NHLS) Central Data Warehouse (CDW) from 2015 to 2019. The NHLS CDW is the national repository for laboratory data from the public health sector of South Africa, serving around 85% of the population. HBV data were extracted by the “Tested_Date” variable for the period 2015 to 2019. Other variables within the HBV dataset included qualitative and quantitative HBsAg and anti-HBc IgM results, gender, date of birth, age, province of testing facility and a CDW allocated unique identification number (UID). A probabilistic linkage algorithm, based on the surname, first name and date of birth or national identification number, was applied to each new entry into the CDW database. If a link to a previous record was identified, the UID was duplicated, otherwise a new UID was generated. Criteria for inclusion in our study were valid HBsAg and anti-HBc IgM qualitative results.

### Data cleaning and deduplication

Data were initially cleaned in Microsoft Excel (Version 2016, Washington, USA) by identification, confirmation and deletion of quality assurance (non-patient related) records and records of research study participants to maintain a database restricted to patient records. Data were then imported into Stata IC (Version 14.1, Texas, USA) for deduplication purposes to exclude test results with duplicate UID records. Deduplication was performed per year from 2015 to 2019. If a patient UID had multiple tests in a specific year, deduplication ensured that a patient appeared only once in that year, providing a more accurate analysis of the data and eliminating risks of over- and under-estimation. Deduplication per year was performed on the complete dataset when determining the total number of cases tested for a specific marker in that year. When determining the total number of cases who tested positive for a specific marker per year, deduplication was performed only after the selection of all positive results for that year, thereby ensuring the inclusion of all cases who tested positive at any point in that year irrespective of testing negative either before and/or after the positive test.

### Statistical analysis

Data were analysed for each year from 2015 to 2019 in Stata IC (version 14.1, Texas, USA) to determine the total number of laboratory-confirmed HBsAg and anti-HBc IgM positive cases by year, and the total number of HBsAg and anti-HBc IgM laboratory tests conducted by year. To prevent over-estimation of anti-HBc IgM positive cases, we excluded anti-HBc IgM values below recommended thresholds for each testing platform in consultation with the NHLS Virology Expert Committee (Supplementary Table 1). Values below the established thresholds were more likely to represent reactivation of HBV infection or chronic flares rather than acute HBV infection [15, 16]. We subsequently calculated the prevalence rates of HBV infection (HBsAg positive cases) per 100,000 population, the incidence rates of acute HBV infection (anti-HBc IgM positive results) per 100,000 population, HBsAg and anti-HBc IgM testing rates per 100,000 population, and HBsAg and anti-HBc IgM test positivity rates. The prevalence rate of HBV infection was calculated as (the number of cases who tested positive for HBsAg × 100,000) / the population estimate. The incidence rate of acute HBV infection was calculated as (the number of cases who tested positive for anti-HBc IgM × 100,000) / the population estimate. HBsAg testing rate was calculated as (the total number of cases tested for HBsAg × 100,000) / the population estimate. Anti-HBc IgM testing rate was calculated as (the total number of cases tested for anti-HBc IgM × 100,000) / the population estimate. HBsAg test positivity rate was calculated as the percentage of HBsAg positive cases over the total number of cases tested for HBsAg. Anti-HBc IgM test positivity rate was calculated as the percentage of anti-HBc IgM positive cases over the total number of cases tested for anti-HBc IgM. Our analysis of HBsAg may reflect a mixture of acute and chronic HBV cases, and amongst those acutely infected, a proportion may have subsequently cleared viral infection.

For prevalence, incidence and testing rates, we used mid-year population estimates as published by Statistics South Africa [39]. Data were analysed per province, gender and age group. Cases with unknown province, gender or age group were included in national totals, however excluded in province-specific, gender-specific and age group-specific analyses respectively.

Statistical analyses were performed on HBsAg and anti-HBc IgM test positivity rates. To analyse trends over time from 2015 to 2019, we used the Kendall’s rank correlation test for time series analysis in Stata IC (Version 14.1, Texas, USA). The Kendall’s rank correlation test generates a correlation coefficient (tau) ranging from − 1 to + 1, where − 0.8 to − 1 is indicative of a strong negative association over time and a + 0.8 to + 1 is indicative of a strong positive association over time. As the correlation coefficient approaches zero, the association weakens. In our study, a negative association indicated a decline in test positivity rates overall from 2015 to 2019, whilst a positive association indicated an increase in test positivity rates overall from 2015 to 2019. To analyse statistical differences between age groups from 2015 to 2019, we used the Mann-Whitney and Kruskal-Wallis tests in GraphPad Prism (Version 9.0.0, California, USA). For all analyses, *p*-values less than 0.05 were considered significant (α level of 0.05). Where the Kruskal-Wallis test resulted in a *p*-value of less than 0.05, the Dunn’s multiple comparison post-hoc test was applied, generating an adjusted p-value based on the number of comparisons.

### Ethics

Ethics approval was obtained from the Institutional Research Ethics Committee (IREC 069/20) of the Durban University of Technology, Durban, South Africa. Approval to obtain the NHLS CDW data was obtained via the NHLS Academic Affairs and Research Management System (PR20254).

## Results

**Fig. 1:**
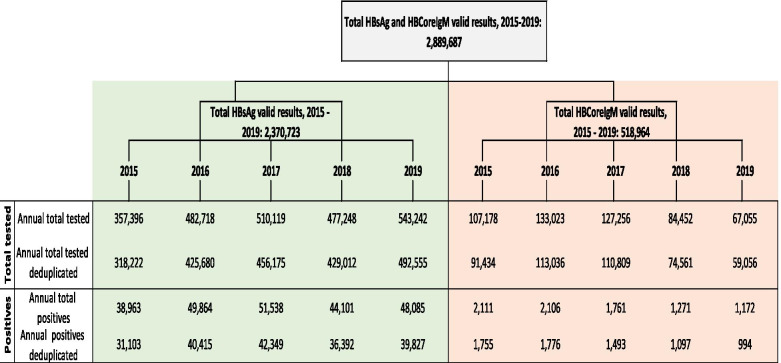
**Hepatitis B sample numbers per year of total tests performed and total positives for HBsAg and anti-HBc IgM prior to and post de-duplication, 2015 to 2019**

For the period 2015 to 2019, the HBV dataset consisted of a total of 2,889,687 records; 2,370,723 valid HBsAg records and 518,964 valid anti-HBc IgM records. Sample numbers per year of total tests performed and total positives for each marker (HBsAg and anti-HBc IgM) prior to and post de-duplication are illustrated in Fig. 1.

### Prevalence of HBV infection

The annual national HBV prevalence rate (HBsAg positive cases per 100,000 population) increased from 56.14 in 2015 to 74.17 in 2017, decreased to 62.81 in 2018 but increased again to 67.76 in 2019 (Supplementary Table 2). This trend was mirrored amongst females and males, with consistently higher prevalence rates in males (Supplementary Table 2). Individuals aged 25 to 49 years had the highest HBV prevalence rates over the 5 year period, with higher rates in males than females (Supplementary Table 2). Amongst individuals 15 to 24 years old, the HBV prevalence rate was substantially lower in individuals 15 to 19 years versus 20 to 24 years, with higher rates in females than males (Supplementary Table 2). Amongst individuals under 15 years of age, the HBV prevalence rate was highest in age group 0 to 4 years, followed by 10 to 14 years and 5 to 9 years, with higher rates in males aged 0 to 4 years, and in females aged 5 to 14 years (Supplementary Table 2). Amongst individuals 50 years and older, HBV prevalence rates decreased by age group, with higher rates in males than females (Supplementary Table 2). Provincially, over the five-year period, the HBV prevalence rate was highest in Gauteng, followed by Eastern Cape and Kwazulu-Natal, and lowest in Northern Cape (Supplementary Table 2).

### HBsAg testing rates

The annual national HBsAg testing rates (total HBsAg tests per 100,000 population) fluctuated over the years, ranging from 574.34 in 2015 to 838.03 in 2019 (Supplementary Table 2). Over the five-year period, individuals aged 20 to 54 years had the highest HBsAg testing rates, higher in females at ages 20 to 44 years, and higher in males at ages 45 to 54 years (Supplementary Table 2). Amongst individuals under 20 years of age, the HBsAg testing rate was highest in age group 15 to 19 years, followed by 10 to 14 years, 0 to 4 years and 5 to 9 years, with higher rates in females than males (Supplementary Table 2). Amongst individuals 55 years and older, the HBsAg testing rate was higher in individuals aged 55 to 59 years than individuals 60 years and older, with higher rates in males than females (Supplementary Table 2). Provincially, over the five-year period, the HBsAg testing rate was highest in Gauteng, followed by Eastern Cape and Kwazulu-Natal, and lowest in Limpopo (Supplementary Table 2).

### HBsAg test positivity rate

**Fig. 2:**
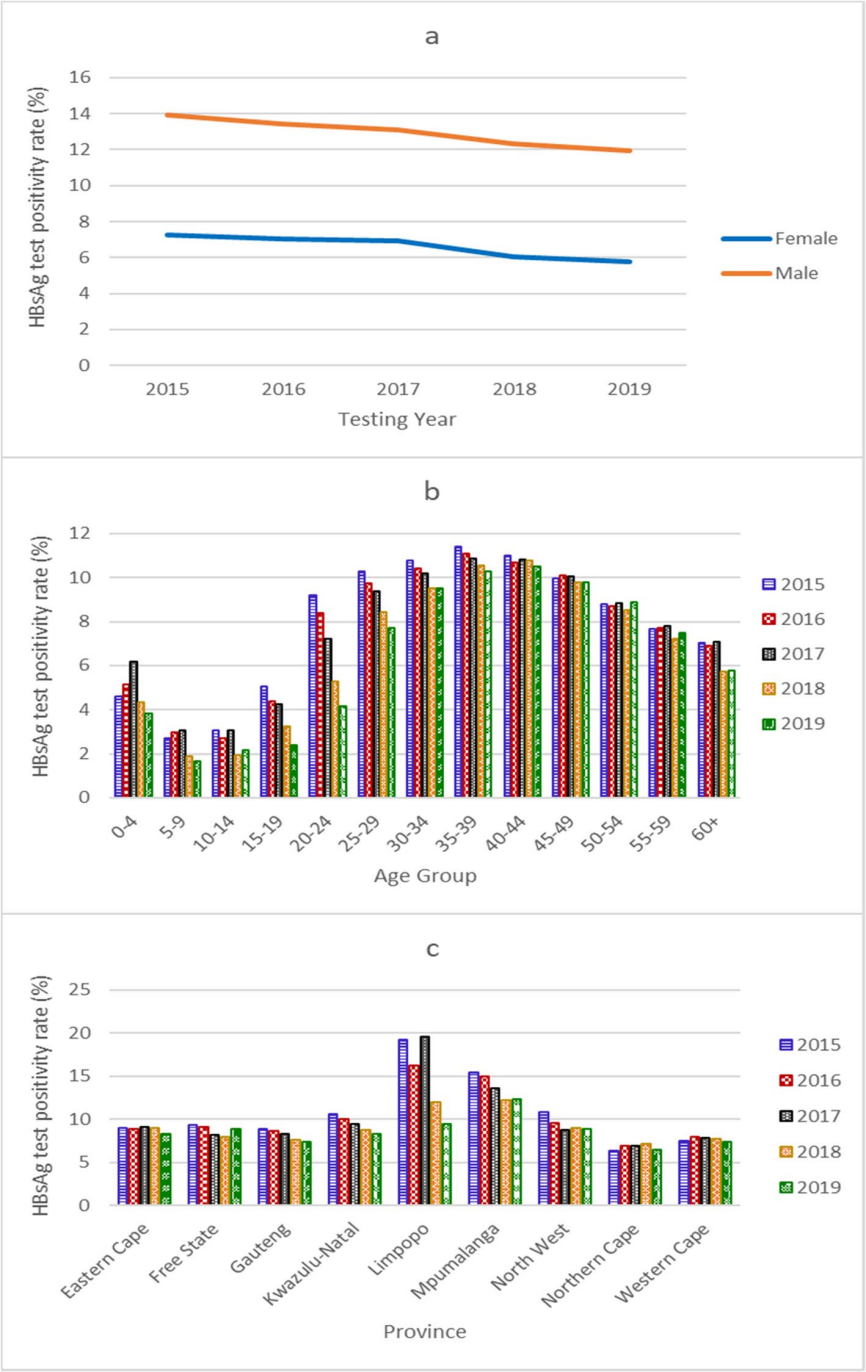
**HBsAg test positivity rate 2015 to 2019 by gender (a), age group (b), and province (c)**. HBsAg test positivity rate was calculated as the percentage of HBsAg positive tests over the total number of HBsAg tests per year

The national HBsAg test positivity rate (percentage of HBsAg positive cases over the total HBsAg tests) declined annually, from 9.77% in 2015 to 8.09% in 2019, with a significant strong negative association over time (tau = − 1.0, *p* = 0.0275), a trend mirrored amongst males and females (Fig. 2a, Supplementary Table 3). Annually, over the five-year period, the HBsAg test positivity rate was significantly higher in males (annual median = 13.09, 95% CI: 11.95 – 13.90) than females (annual median = 6.90, 95% CI: 5.78 – 7.23) (*p* = 0.0079) (Fig. 2a, Supplementary Table 3).

Individuals aged 25 to 49 years had the highest HBsAg test positivity rates (Fig. 2b, Supplementary Table 3). Amongst individuals 50 years and older, the HBsAg test positivity rate decreased by age group (Supplementary Table 3). Amongst individuals 15 to 24 years old, the HBsAg test positivity rate was lower in individuals 15 to 19 years (annual median = 4.25, 95% CI: 2.39-5.04) than 20 to 24 years (annual median = 7.20, 95% CI: 4.17-9.18) (Supplementary Table 3). In children under 15 years of age, the HBsAg test positivity rate was significantly higher in children aged 0 to 4 years (annual median = 4.60, 95% CI: 3.82 – 6.16) than children aged 5 to 9 years (annual median = 2.72, 95% CI: 1.67 – 3.08) and 10 to 14 years (annual median = 2.71, 95% CI: 1.94 – 3.08, *p* = 0.0104 and 0.0240 respectively). In children under 5 years of age who tested positive for HBsAg (*n* = 1131), 65.7% (743) were under 1 year old (Table 1). The majority of cases under 1 year old were from Gauteng (221/743, 29.74%), followed by Limpopo (133/743, 17.90%) and Kwazulu-Natal (129/743, 17.36%) (Table 1).

**Table 1:** Age distribution of HBsAg positive cases under 5 years old by province, 2015 to 2019

Province	Age in years						Percent of Total (%)
	0	1	2	3	4	Total (No)	
Eastern Cape	74	12	7	5	9	107	9.46
Free State	13	3	2	0	3	21	1.86
Gauteng	221	24	20	16	22	303	26.79
Kwazulu-Natal	129	27	11	12	20	199	17.60
Limpopo	133	31	31	34	27	256	22.63
Mpumalanga	62	10	8	6	6	92	8.13
North West	32	2	2	1	2	39	3.45
Northern Cape	10	0	0	3	2	15	1.33
Western Cape	69	11	11	3	5	99	8.75
**Total**	**743**	**120**	**92**	**80**	**96**	**1131**	**100**

The HBsAg test positivity rate was higher in males in all age groups than females, significantly higher in males than females 25 years and older (p = 0.0079) (Supplementary Table 3). Trend analysis over time showed a significant strong negative association in age groups 15 to 19 years, 20 to 24 years, 25 to 29 years and 35 to 39 years (tau = − 1.0, *p* = 0.0275), and a strong negative association in age group 30 to 34 years (tau = − 0.8, *p* = 0.0864) (Supplementary Table 3).

Amongst females of child-bearing age (15 to 49 years) over the five years, the median HBsAg test positivity rate was 7.18%, declining from 7.63% in 2015 to 6.69% in 2019 (Supplementary Table 3). Provincially, over the five years, the HBsAg test positivity rate was notably higher in Limpopo and Mpumalanga, and lowest in Northern Cape (Fig. 2c, Supplementary Table 3). The HBsAg test positivity rate in Limpopo dropped markedly in 2018 and in 2019, with a moderate negative association over time (tau = − 0.6, *p* = 0.2207). Trend analysis of the HBsAg test positivity rates over time showed a significant strong negative association in Gauteng and Kwazulu-Natal provinces (tau = − 1.0, *p* = 0.0275), and a strong negative association in Mpumalanga province (tau = − 0.8, *p* = 0.0864) (Fig. 2c, Supplementary Table 3).

### Incidence of acute HBV infection

**Fig. 3:**
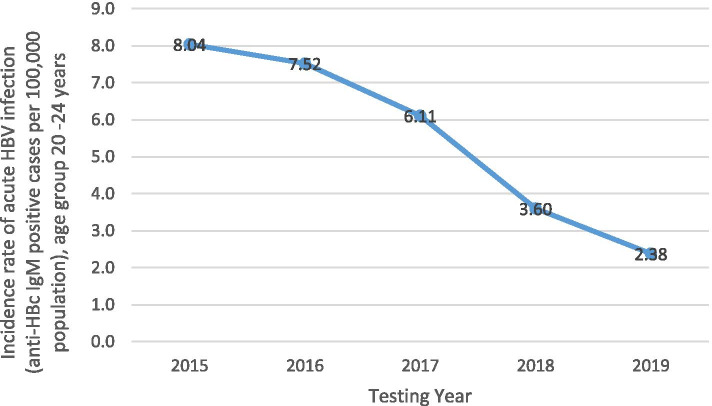
**National incidence rate of acute HBV infection per 100,000 population amongst individuals 20 to 24 years old, 2015 to 2019.** Incidence rate was calculated as (the number of cases who tested positive for anti-HBc IgM × 100,000) / the population estimate per year

The annual national incidence rate of acute HBV infection (anti-HBc IgM positive cases per 100,000 population) dropped from 3.17 in 2015 to 1.69 in 2019 (Supplementary Table 4). This decline was largely influenced by the decline in incidence rates amongst individuals 20 to 24 years old, from 8.04 per 100,000 population in 2015 to 2.38 per 100,000 population in 2019 (Fig. 3). In males, there was a decline in the incidence rates over the years; however, in females the rate peaked at 3.35 per 100,000 population in 2016, declining thereafter (Supplementary Table 4). Individuals 20 to 39 years old had the highest incidence rates over the five years, higher in females at ages 20 to 29 years and higher in males at ages 30 to 39 years (Supplementary Table 4). Amongst individuals under 20 years of age, the incidence rate was higher amongst individuals aged 15 to 19 years than individuals aged 0 to 14 years, higher in females 15 to 19 years old and similar between males and females 0 to 14 years old (Supplementary Table 4). Amongst individuals 40 years and older, incidence rates decreased by age group, with higher rates in males than females (Supplementary Table 4).

Provincially, over the five years, the incidence rate of acute HBV infection was highest in Mpumalanga, followed by Kwazulu-Natal and Gauteng, and lowest in Limpopo (Supplementary Table 4). We saw noteworthy declines in the incidence rates per 100,000 population in Eastern Cape province from 2.58 in 2015 to 1.06 in 2019, and in Mpumalanga province from 3.99 in 2015 to 1.11 in 2019 (Supplementary Table 4).

### Anti-HBc IgM testing rate

The anti-HBc IgM testing rate (total anti-HBc IgM tests per 100,000 population) peaked in 2016, but dropped thereafter (Supplementary Table 4). Over the five years, individuals aged 25 to 54 years had the highest anti-HBc IgM testing rates, higher in females at ages 25 to 39 years and higher in males at ages 40 to 54 years (Supplementary Table 4). Amongst individuals 15 to 24 years of age, the anti-HBc IgM testing rate was substantially higher in individuals aged 20 to 24 years than individuals aged 15 to 19 years, with higher rates in females than males (Supplementary Table 4). Amongst individuals under 15 years of age, the anti-HBc IgM testing rate was highest in age group 10 to 14 years, followed by age group 0 to 4 years and age group 5 to 9 years, with higher rates in females than males (Supplementary Table 4). Amongst individuals 55 years and older, the anti-HBc IgM testing rate was higher in age group 55 to 59 years than individuals 60 years and older, higher in males than females (Supplementary Table 4). Provincially, over the five years, the anti-HBc IgM testing rate was highest in Northern Cape, followed by Eastern Cape and Free State, and lowest in Mpumalanga (Supplementary Table 4). We noted a substantial decline in the anti-HBc IgM testing rate per 100,000 population in Eastern Cape province from 329.25 in 2015 to 24.36 in 2019 (Supplementary Table 4).

### Anti-HBc IgM test positivity rate

**Fig. 4:**
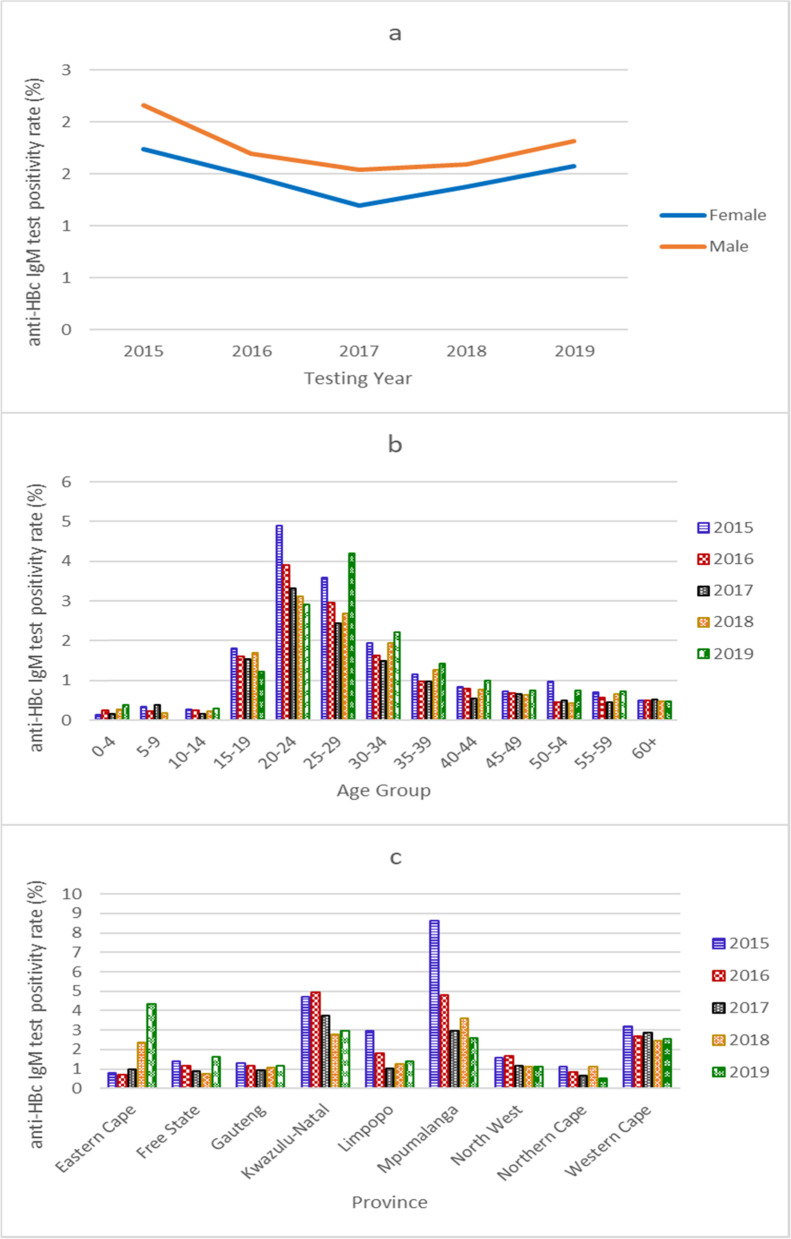
**Anti-HBc IgM test positivity rate 2015 to 2019 by gender (a), age group (b), and province (c).** Anti-HBc IgM test positivity rate was calculated as the percentage of anti-HBc IgM positive cases over the total anti-HBc IgM tests

The national anti-HBc IgM test positivity rate (percentage of anti-HBc IgM positive cases over the total anti-HBc IgM tests) reached a low point of 1.35% in 2017, subsequently increasing to 1.68% in 2019 (Fig. 4a, Supplementary Table 5). Trend analysis showed a weak negative association in the national and gender-specific anti-HBc IgM test positivity rate over time (tau = − 0.2, *p* = 0.8065, Supplementary Table 5). There was no difference between males and females in the anti-HBc IgM test positivity rates over the five years (Supplementary Table 5). Individuals aged 15 to 39 years had the highest anti-HBc IgM test positivity rates, significantly higher in females 15 to 19 years old and in males 20 to 24 years, 25 to 29 years, and 30 to 34 years old (Fig. 4b, Supplementary Table 5).

Amongst individuals under 15 years of age, the anti-HBc IgM test positivity rate was similar in age groups 0 to 4 years (annual median = 0.25, 95% CI: 0.15-0.38), 5 to 9 years (annual median = 0.24, 95% CI: 0.00-0.38) and 10 to 14 years (annual median = 0.26, 95% CI: 0.17-0.30, overall *p* = 0.9803), and slightly higher in males (Supplementary Table 5). Amongst individuals 40 years and older, the anti-HBc IgM test positivity rates were similar amongst all age groups, as well as between females and males (Supplementary Table 5). Trend analysis of the anti-HBc IgM test positivity rates in all age groups over time showed a significant negative association in age group 20 to 24 years (tau = − 1.0, *p* = 0.0275) (Supplementary Table 5). Provincially, over the five years, the anti-HBc IgM test positivity rate was highest in Kwazulu-Natal, followed by Mpumalanga and Western Cape, and lowest in Northern Cape (Supplementary Table 5, Fig. 4c). We saw a noteworthy drop in the anti-HBc IgM test positivity rate in Mpumalanga province from 8.62% in 2015 to 2.57% in 2019, with a strong negative association over time (tau = − 0.8, *p* = 0.0864). On the contrary, we noted a concerning rise in the anti-HBc IgM test positivity rate in Eastern Cape province, from 0.79% in 2015 to 4.34% in 2019, with a strong positive association over time (tau = + 0.8, p = 0.0864) (Supplementary Table 5, Fig. 4c).

## Discussion

We analysed countrywide hepatitis B prevalence, incidence and test positivity rates annually for five years from 2015 to 2019 from laboratory-confirmed HBV tests conducted in the NHLS. The national HBV prevalence and HBsAg testing rates per 100,000 population fluctuated over the years; however, the HBsAg test positivity rate showed a consistent downward trend. Similarly, there was a decline in the national incidence rate of acute HBV infection per 100,000 population over the years. The downward trend in the national HBsAg test positivity rates and the incidence rates of acute HBV infection are most likely attributed to the inclusion of the HBV vaccine into the routine EPI schedule in 1995, and suggest that although HBV transmission is ongoing, HBV disease prevalence and incidence is slowly declining. A decline in the HBsAg seroprevalence from 12.8% in 1995 to 3.0% in 2009 has previously been reported amongst children under five years old in South Africa [17, 28]. Globally, reductions in the HBV incidence rates and HBsAg seroprevalence rates have also been reported in many other countries and regions of the world following the initiation of universal HBV vaccination [28, 32, 40].

The HBV prevalence and HBsAg test positivity rates were substantially higher in males. However, incidence rates of acute HBV infection and anti-HBc IgM test positivity rates were comparable between males and females. A plausible explanation is that despite similar rates of acute HBV infections, more males progress to chronic disease, consistent with reviews of gender differences in response to HBV infection and with findings of studies conducted in Greece, New Zealand and Taiwan [41–44]. With regards to testing rates, females had higher rates overall, however testing patterns varied with age. Whilst females had substantially higher rates at earlier ages, males had higher rates at older ages. The higher testing rates in females at earlier ages are likely due to the higher probability of symptomatic infection in females due to their more intense inflammatory responses to viral infections, coupled with more frequent healthcare seeking behaviour, including visits during pregnancy and child-care visits [45, 46]. As a result, there may be a considerable proportion of undiagnosed acute HBV infection in males. The higher testing rates in males at older ages is likely due to their increased risk of persistent HBV infection and progression to chronicity, with subsequent reactivation of infection or chronic flares warranting healthcare seeking behaviour at these ages [41–43, 45].

HBV prevalence and HBsAg test positivity rates were highest amongst individuals aged 25 to 49 years, which is not surprising as persons above 24 years would not have been eligible for the HBV vaccination programme as infants. Although our cross-sectional approach did not distinguish between transiently positive HBsAg results and results that remained positive for more than six months, it is likely that a substantial proportion of individuals positive for HBsAg are/were chronic carriers and therefore a possible source of HBV transmission [19]. Regarding acute HBV infections, we saw that individuals 20 to 39 years old had the highest incidence rates per 100,000 population, and individuals 15 to 39 years old had the highest anti-HBc IgM test positivity rates. These distributions suggest risky lifestyle habits amongst individuals 15 to 39 years old, including multiple sexual partners, unprotected sex and/or intravenous drug use, resulting in increased HBV infection rates at these ages [47–50]. In individuals 20 to 24 years old, a striking observation was the substantial decline in the incidence rates of acute HBV infection, HBsAg and anti-HBc IgM test positivity rates over the years. In 2015, only individuals aged 20 years were vaccine eligible as infants, while by 2019 all individuals within the age group of 20 to 24 years were vaccine eligible as infants. The decline in the annual incidence rates of acute HBV infection amongst individuals 20 to 24 years old demonstrates the positive impact of the HBV vaccination programme, even with suboptimal vaccine coverage rates [51]. Interestingly, we also noted declining HBsAg test positivity rates in age groups 25 to 29, 30 to 34 and 35 to 39 years, which suggest reduction in transmission of HBV amongst individuals who were not vaccine eligible, possibly due to reduced transmission in the community overall.

The HBV prevalence and HBsAg test positivity rates amongst individuals 50 years and older may be linked to the higher probability of symptomatic chronic HBV infection at these ages, leading to healthcare-seeking behaviour. However, incidence rates of acute HBV infection and anti-HBc IgM test positivity rates observed amongst individuals 50 years and older indicate ongoing transmission of HBV infection at these older ages.

Amongst children under 15 years of age, ages least likely to be associated with sexual behavior and intravenous drug use, the HBV prevalence and HBsAg test positivity rates were higher in children 0 to 4 years old versus age groups of 5 to 9 years and 10 to 14 years. Further analysis of the number of HBsAg positive cases under 5 years old showed that the bulk of the cases were under 1 year old. As South Africa has not yet implemented a birth dose of the HBV vaccine into its routine EPI schedule, and a considerable proportion of females of child-bearing age tested positive for HBsAg, HBV infection in infants is more likely associated with vertical rather than horizontal transmission. Also, although HBV testing in pregnancy is recommended by the South African national Hepatitis B guidelines, it is not widely implemented. A possible confounder of HBsAg positivity in cases under 1 year old may be attributed to transient HBsAg positivity following HBV vaccination [52]. However, as hepatitis B is usually described as subclinical in infants and young individuals, the reasons for HBsAg testing in at these ages are uncertain and cannot be determined without access to their or their mother’s clinical histories. Reasons for testing may include symptomatic cases, infants admitted for other medical conditions in which HBV screening was performed, or screening of infants born to mothers known to be HBV infected. Infants infected from their mothers may also be diagnosed with HBV infection at much later ages [53]. Vertical transmission may be significantly reduced with inclusion of a HepB_BD into the current EPI schedule, subsequently minimising the national burden of chronic HBV infection [54]. Amongst individuals 5 to 14 years old, the low HBV prevalence and HBsAg test positivity rates may reflect the subclinical phase of HBV infection at these ages and/or effective vaccination within this group. However, HBV infection in children and teenagers can still occur where there is suboptimal vaccine coverage, resulting in continued transmission in these populations. Suboptimal vaccine efficacy in risk groups, such as HIV-infected children, may also be a contributing factor [55].

HBV prevalence and HBsAg testing rates were substantially higher in Gauteng province compared with other provinces. Gauteng is the economic hub of South Africa with numerous excellent health facilities. Individuals may have travelled from other provinces to Gauteng for healthcare purposes. The HBsAg test positivity rate, however, was highest in Limpopo province, declining markedly over time, while the HBV prevalence rate in Limpopo was quite low in comparison to other provinces. The reasons for high HBsAg test positivity rates may therefore reflect HBsAg testing practices, although test positivity rates may reflect disease burden [56, 57]. We therefore regard the high HBsAg test positivity rate in Limpopo province as indicative of a particularly high disease burden which declined over time. Regarding incidence rates of acute HBV infection and anti-HBc IgM test positivity rates over the five years, the two provinces with the highest rates were Mpumalanga and Kwazulu-Natal. In Mpumalanga, there was a substantial decline in the incidence and anti-HBc IgM test positivity rates from 2015 to 2019. In Eastern Cape, there was a substantial increase in the anti-HBc IgM test positivity rate from 2015 to 2019, with a substantial decrease in the incidence rates of acute HBV infection and anti-HBc IgM testing rates. Changes to the anti-HBc IgM testing practices, particularly in Mpumalanga and Eastern Cape provinces, may have contributed to the change in rates over time. Given these findings and provincial disparities within South Africa, further investigations to better understand risk behaviour, testing practices and vaccination coverage, are warranted in Limpopo, Mpumalanga, Eastern Cape and Kwazulu-Natal provinces [58]. Where applicable, targeted provincial interventions would subsequently decrease countrywide incidence of HBV infection.

Passive surveillance has the limitation of only detecting laboratory-confirmed cases, and we cannot extrapolate our data to estimate community prevalence or incidence of undiagnosed HBV infection. Such estimation would require random sampling of households with blood draws. We did not use estimates of health seeking behaviour to estimate numbers of undiagnosed cases. The reported province reflects the location of the testing facility and not necessarily the place of residence or birth for each case. People may have travelled to preferred facilities or as referrals for enhanced care and management. In addition, due to limited availability of diagnostic testing amongst public health facilities in South Africa, and variation in access and utilization of testing by province, the numbers in provincial differences presented here may represent differences in testing practices rather than disease burden. As testing for anti-HBc IgM is usually associated with symptomatic manifestations of HBV infection, an increased anti-HBc IgM test positivity rate may suggest that more clinically suspected acute HBV infections were laboratory confirmed rather than an increase in the acute infection rate, however experience with other diseases has shown the value of monitoring trends in test positivity rates over time. By excluding anti-HBc IgM positive results with values below an established threshold, we may have excluded a proportion of true acute HBV infections in our analyses. Data entry errors in patient identification details may have affected the NHLS CDW linkage process, allocating a new UID to an existing case. Deduplication exercises were performed per year and not collectively over the five-year period. We therefore did not exclude individuals who were tested in multiple years and have reported our data as annual rates, an approach that is reproducible over time. A strength of our study, however, is analyses of a mega dataset of 2,889,687 records of tests conducted in the public health sector of South Africa, which serves around 85% of the national population.

## Conclusion

We conclude that thorough interrogation of passive laboratory data, not exclusive to HBV, is an informative resource and may be valuable for planning public health programmes or serve as evidence or a reference point for long-term public health interventions. Optimal vaccination coverage at 6, 10, and 14 week visits and interruption of vertical transmission is critical to hepatitis B elimination. Tools to minimise vertical transmission of HBV are inclusion of HepB_BD vaccine into the routine EPI schedule of South Africa, or nation-wide implementation of screening of mothers for HBV to mitigate associated risks. As new generations of vaccinated infants and children reach adulthood, hepatitis B incidence and prevalence should shift to older age groups, subsequently declining in younger individuals, including women of reproductive age. Without vaccination programmes in adults, hepatitis B incidence may be reduced through heightened awareness of transmission routes and prevention measures.

## Supplementary Information

**Additional file 1.**

**Additional file 2.**

**Additional file 3.**

**Additional file 4.**

**Additional file 5.**

## Data Availability

The datasets used and/or analysed during the current study are available from the corresponding author on reasonable request.

## Abbreviations

HBV: Hepatitis B virus
HBsAg: Hepatitis B surface antigen
anti-HBc IgM: IgM antibody to the hepatitis B core antigen
WHO: World Health Organisation
EPI: Expanded Programme on Immunisation
HepB3: Hepatitis B vaccine administered at 6, 10 and 14 weeks of age
HepB_BD: Birth dose of the hepatitis B vaccine
NHLS: National Health Laboratory Service
CDW: Central Data Warehouse
UID: Unique identification
