# Supplementary material for: Prevalence and incidence rates of laboratory-confirmed hepatitis B infection in South Africa, 2015 to 2019
Source: BMC Public Health. 2022 Jan 6;22:29. doi: 10.1186/s12889-021-12391-3 (PMC8739689; doi:10.1186/s12889-021-12391-3)
Supplement: Supplementary file 1 — Additional file 1. [file 12889_2021_12391_MOESM1_ESM.pdf]

Supplementary Table 1: Anti-HBc IgM thresholds for identification of acute hepatitis B cases

Instrument platform	Anti-HBc IgM values indicative of a positive result	Recommended threshold*
Abbott Architect	1 to 50	20
Siemens Advia Centaur	1 to >9	≥9
Beckman DXI	1 to 24	5
Roche Cobas	1 to 50	20
DiaSorin Liason	15 to 34	20

*Anti-HBc IgM thresholds recommended by the Virology Expert Committee of the NHLS. Anti-HBc IgM values below the recommended thresholds were excluded from analyses of acute HBV cases as they were more likely to represent reactivation of infection or flares amongst chronic carriers
