# Supplementary material for: Prevalence and incidence rates of laboratory-confirmed hepatitis B infection in South Africa, 2015 to 2019
Source: BMC Public Health. 2022 Jan 6;22:29. doi: 10.1186/s12889-021-12391-3 (PMC8739689; doi:10.1186/s12889-021-12391-3)
Supplement: Supplementary file 2 — Additional file 2. [file 12889_2021_12391_MOESM2_ESM.pdf]

Supplementary Table 2: Prevalence of HBV infection and HBsAg testing rates, 2015 to 2019

Testing Year	Prevalence (HBsAg positive cases) per 100,000 population								HBsAg testing rate per 100,000 population							
	2015	2016	2017	2018	2019	2015 - 2019			2015	2016	2017	2018	2019	2015 - 2019		
						Median	Lower 95% CI	Upper 95% CI						Median	Lower 95% CI	Upper 95% CI
**Annual**	56.14	71.85	74.17	62.81	67.76	**67.76**	56.14	74.17	574.34	756.73	798.94	740.45	838.03	**756.73**	574.34	838.03
**Female**	49.33	63.69	65.56	53.06	58.14	**58.14**	49.33	65.56	682.18	902.25	950.22	875.55	1005.88	**902.25**	682.18	1005.88
**Male**	61.48	78.14	81.06	71.13	75.26	**75.26**	61.48	81.06	442.25	582.87	619.37	577.04	629.74	**582.87**	442.25	629.74
**Age Group 0-4**	3.66	4.27	5.10	3.61	3.23	**3.66**	3.23	5.10	79.45	83.19	82.80	83.70	84.39	**83.19**	79.45	84.39
Female	3.02	3.90	4.95	3.15	2.83	**3.15**	2.83	4.95	76.56	85.46	83.80	82.21	82.49	**82.49**	76.56	85.46
Male	4.07	4.31	5.13	3.86	3.55	**4.07**	3.55	5.13	76.57	76.22	77.32	80.67	80.63	**77.32**	76.22	80.67
**Age Group 5-9**	1.69	1.91	1.95	1.08	0.98	**1.69**	0.98	1.95	62.19	63.83	63.32	57.92	58.51	**62.19**	57.92	63.83
Female	1.60	1.86	1.92	1.06	0.88	**1.60**	0.88	1.92	68.92	71.76	70.64	66.86	66.64	**68.92**	66.64	71.76
Male	1.57	1.93	1.87	1.00	1.03	**1.57**	1.00	1.93	52.04	53.04	53.99	46.15	47.34	**52.04**	46.15	53.99
**Age Group 10-14**	2.91	2.99	3.49	2.05	2.40	**2.91**	2.05	3.49	94.25	110.49	114.72	105.73	111.44	**110.49**	94.25	114.72
Female	3.74	3.47	3.59	2.57	2.86	**3.47**	2.57	3.74	115.86	142.10	146.27	139.29	146.47	**142.10**	115.86	146.47
Male	2.05	2.48	3.32	1.43	1.75	**2.05**	1.43	3.32	69.68	76.00	79.80	69.77	72.23	**72.23**	69.68	79.80
**Age Group 15-19**	11.62	13.57	14.29	10.61	11.67	**11.67**	10.61	14.29	230.68	310.69	336.21	327.70	489.21	**327.70**	230.68	489.21
Female	16.81	21.15	21.07	14.71	16.19	**16.81**	14.71	21.15	352.52	478.84	514.83	498.74	765.84	**498.74**	352.52	765.84
Male	6.29	5.64	7.29	6.30	6.70	**6.30**	5.64	7.29	103.14	135.49	151.41	149.24	187.97	**149.24**	103.14	187.97
**Age Group 20-24**	52.97	65.54	60.68	43.03	44.87	**52.97**	43.03	65.54	576.92	779.75	842.94	818.15	1077.00	**818.15**	576.92	1077.00
Female	69.35	88.63	81.26	58.01	63.06	**69.35**	58.01	88.63	908.89	1235.65	1335.43	1284.20	1689.03	**1284.20**	908.89	1689.03
Male	35.04	40.62	39.01	27.01	25.13	**35.04**	25.13	40.62	235.48	311.19	337.14	334.82	428.78	**334.82**	235.48	428.78
**Age Group 25-29**	90.76	116.52	118.16	99.81	105.60	**105.60**	90.76	118.16	882.05	1195.29	1258.85	1185.89	1372.69	**1195.29**	882.05	1372.69
Female	100.64	129.96	133.84	114.89	120.61	**120.61**	100.64	133.84	1255.16	1716.84	1815.07	1710.18	1968.75	**1716.84**	1255.16	1968.75
Male	78.36	99.84	99.69	82.39	86.79	**86.79**	78.36	99.84	498.40	658.98	692.49	646.14	746.20	**658.98**	498.40	746.20
**Age Group 30-34**	119.92	154.02	159.27	131.94	143.70	**143.70**	119.92	159.27	1113.15	1479.50	1561.27	1385.10	1508.19	**1479.50**	1113.15	1561.27
Female	111.69	145.03	155.64	122.28	132.37	**132.37**	111.69	155.64	1422.49	1885.92	2003.72	1767.42	1933.66	**1885.92**	1422.49	2003.72
Male	125.32	158.67	158.99	137.56	149.08	**149.08**	125.32	158.99	789.97	1055.59	1105.22	983.96	1052.32	**1052.32**	789.97	1105.22
**Age Group 35-39**	133.64	170.81	174.06	155.00	163.96	**163.96**	133.64	174.06	1172.69	1541.31	1603.30	1470.01	1597.05	**1541.31**	1172.69	1603.30
Female	103.84	132.70	141.59	118.87	132.41	**132.41**	103.84	141.59	1321.33	1751.65	1836.13	1671.59	1842.51	**1751.65**	1321.33	1842.51
Male	160.20	204.60	201.84	185.90	190.17	**190.17**	160.20	204.60	997.14	1300.13	1337.80	1238.04	1310.26	**1300.13**	997.14	1337.80
**Age Group 40-44**	117.46	154.29	165.09	146.97	153.46	**153.46**	117.46	165.09	1068.59	1445.49	1528.49	1360.90	1459.22	**1445.49**	1068.59	1528.49
Female	82.12	109.11	111.06	99.84	108.31	**108.31**	82.12	111.06	1111.90	1483.49	1546.81	1364.10	1503.61	**1483.49**	1111.90	1546.81
Male	152.20	197.62	217.97	192.40	194.36	**194.36**	152.20	217.97	1000.30	1375.45	1477.29	1323.97	1369.68	**1369.68**	1000.30	1477.29
**Age Group 45-49**	89.17	121.51	127.10	113.84	120.83	**120.83**	89.17	127.10	893.37	1201.69	1266.06	1163.85	1236.56	**1201.69**	893.37	1266.06
Female	58.24	80.42	81.83	69.08	79.54	**79.54**	58.24	81.83	869.98	1164.59	1222.25	1112.10	1189.00	**1164.59**	869.98	1222.25
Male	123.92	166.05	174.65	161.11	162.44	**162.44**	123.92	174.65	900.52	1219.17	1291.61	1192.13	1251.44	**1219.17**	900.52	1291.61
**Age Group 50-54**	66.39	87.09	92.21	80.23	90.81	**87.09**	66.39	92.21	754.14	1000.13	1041.77	941.27	1022.31	**1000.13**	754.14	1041.77
Female	45.79	62.72	63.87	51.20	58.14	**58.14**	45.79	63.87	737.30	965.14	980.81	880.40	958.05	**958.05**	737.30	980.81
Male	90.17	116.32	126.22	114.96	128.24	**116.32**	90.17	128.24	756.79	1023.15	1098.54	995.06	1073.46	**1023.15**	756.79	1098.54
**Age Group 55-59**	46.53	61.32	65.17	56.63	63.76	**61.32**	46.53	65.17	605.72	793.80	837.11	786.49	851.72	**793.80**	605.72	851.72
Female	31.40	42.82	42.08	36.08	41.13	**41.13**	31.40	42.82	570.57	736.24	769.77	719.86	790.78	**736.24**	570.57	790.78
Male	65.29	84.06	94.15	82.26	91.81	**84.06**	65.29	94.15	637.61	849.48	907.81	856.70	906.19	**856.70**	637.61	907.81
**Age Group 60+**	26.14	31.33	34.05	27.68	29.57	**29.57**	26.14	34.05	372.70	454.14	479.82	482.93	513.19	**479.82**	372.70	513.19
Female	17.97	21.35	24.65	18.03	18.95	**18.95**	17.97	24.65	322.94	394.72	412.77	412.90	442.71	**412.77**	322.94	442.71
Male	38.13	45.78	47.46	41.84	45.37	**45.37**	38.13	47.46	439.27	535.80	574.10	579.63	606.22	**574.10**	439.27	606.22
**Province**																
Eastern Cape	61.66	72.39	80.41	72.91	70.86	**72.39**	61.66	80.41	689.35	813.35	881.39	813.92	859.04	**813.92**	689.35	881.39
Free State	56.28	66.51	59.52	50.49	67.08	**59.52**	50.49	67.08	603.16	734.48	731.19	636.06	760.87	**731.19**	603.16	760.87
Gauteng	74.74	106.75	108.55	89.62	101.40	**101.40**	74.74	108.55	838.80	1237.12	1313.98	1175.42	1381.69	**1237.12**	838.80	1381.69
Kwazulu-Natal	67.82	77.17	67.81	63.91	71.16	**67.82**	63.91	77.17	636.60	773.99	720.16	734.16	859.38	**734.16**	636.60	859.38
Limpopo	33.74	41.75	53.82	28.01	20.59	**33.74**	20.59	53.82	175.27	256.72	275.67	234.88	216.80	**234.88**	175.27	275.67
Mpumalanga	43.46	69.40	87.96	63.56	66.61	**66.61**	43.46	87.96	282.05	464.96	648.54	519.32	538.35	**519.32**	282.05	648.54
North West	33.39	49.79	53.01	57.91	63.72	**53.01**	33.39	63.72	308.56	523.72	602.98	646.98	719.42	**602.98**	308.56	719.42
Northern Cape	30.12	31.29	30.90	33.64	31.02	**31.02**	30.12	33.64	471.21	452.74	446.62	467.40	483.43	**467.40**	446.62	483.43
Western Cape	37.55	38.13	38.97	35.62	36.00	**37.55**	35.62	38.97	501.62	476.42	498.27	464.63	488.69	**488.69**	464.63	501.62
