# Supplementary material for: Prevalence and incidence rates of laboratory-confirmed hepatitis B infection in South Africa, 2015 to 2019
Source: BMC Public Health. 2022 Jan 6;22:29. doi: 10.1186/s12889-021-12391-3 (PMC8739689; doi:10.1186/s12889-021-12391-3)
Supplement: Supplementary file 3 — Additional file 3. [file 12889_2021_12391_MOESM3_ESM.pdf]

Supplementary Table 3: Annual HBsAg test positivity rates by gender, age group and province, 2015 to 2019

Testing Year	2015 (%)	2016 (%)	2017 (%)	2018 (%)	2019 (%)	2015 - 2019					
						**Median (%)**	Lower 95% CI	Upper 95% CI	Kendall's tau[a]	Kendall's p-value[a]	Mann-Whitney p-value[b]
**Annual**	9.77	9.49	9.28	8.48	8.09	**9.28**	8.09	9.77	-1.0	**0.0275***	-
**Female**	7.23	7.06	6.90	6.06	5.78	**6.90**	5.78	7.23	-1.0	**0.0275***	**0.0079***
**Male**	13.90	13.41	13.09	12.33	11.95	**13.09**	11.95	13.90	-1.0	**0.0275***	
**Age Group 0-4**	4.60	5.13	6.16	4.32	3.82	**4.60**	3.82	6.16	-0.4	0.4624	-
Female	3.94	4.56	5.91	3.83	3.43	**3.94**	3.43	5.91	-	-	0.1508
Male	5.32	5.66	6.64	4.78	4.40	**5.32**	4.40	6.64	-	-	
**Age Group 5-9**	2.72	3.00	3.08	1.87	1.67	**2.72**	1.67	3.08	-0.4	0.4624	-
Female	2.32	2.59	2.72	1.59	1.33	**2.32**	1.33	2.72	-	-	0.2222
Male	3.01	3.63	3.47	2.17	2.18	**3.01**	2.17	3.63	-	-	
**Age Group 10-14**	3.08	2.71	3.05	1.94	2.15	**2.71**	1.94	3.08	-0.6	0.2207	-
Female	3.23	2.44	2.45	1.84	1.95	**2.44**	1.84	3.23	-	-	0.3095
Male	2.94	3.26	4.17	2.05	2.43	**2.94**	2.05	4.17	-	-	
**Age Group 15-19**	5.04	4.37	4.25	3.24	2.39	**4.25**	2.39	5.04	-1.0	**0.0275***	-
Female	4.77	4.42	4.09	2.95	2.11	**4.09**	2.11	4.77	-	-	0.3095
Male	6.09	4.17	4.81	4.22	3.56	**4.22**	3.56	6.09	-	-	
**Age Group 20-24**	9.18	8.41	7.20	5.26	4.17	**7.20**	4.17	9.18	-1.0	**0.0275***	-
Female	7.63	7.17	6.08	4.52	3.73	**6.08**	3.73	7.63	-	-	0.0556
Male	14.88	13.05	11.57	8.07	5.86	**11.57**	5.86	14.88	-	-	
**Age Group 25-29**	10.29	9.75	9.39	8.42	7.69	**9.39**	7.69	10.29	-1.0	**0.0275***	-
Female	8.02	7.57	7.37	6.72	6.13	**7.37**	6.13	8.02	-	-	**0.0079***
Male	15.72	15.15	14.40	12.75	11.63	**14.40**	11.63	15.72	-	-	
**Age Group 30-34**	10.77	10.41	10.20	9.53	9.53	**10.20**	9.53	10.77	-0.8	0.0864	-
Female	7.85	7.69	7.77	6.92	6.85	**7.69**	6.85	7.85	-	-	**0.0079***
Male	15.86	15.03	14.39	13.98	14.17	**14.39**	13.98	15.86	-	-	
**Age Group 35-39**	11.40	11.08	10.86	10.54	10.27	**10.86**	10.27	11.40	-1.0	**0.0275***	-
Female	7.86	7.58	7.71	7.11	7.19	**7.58**	7.11	7.86	-	-	**0.0079***
Male	16.07	15.74	15.09	15.02	14.51	**15.09**	14.51	16.07	-	-	
**Age Group 40-44**	10.99	10.67	10.80	10.80	10.52	**10.80**	10.52	10.99	-0.6	0.2207	-
Female	7.39	7.35	7.18	7.32	7.20	**7.32**	7.18	7.39	-	-	**0.0079***
Male	15.22	14.37	14.75	14.53	14.19	**14.53**	14.19	15.22	-	-	
**Age Group 45-49**	9.98	10.11	10.04	9.78	9.77	**9.98**	9.77	10.11	-0.6	0.2207	-
Female	6.69	6.91	6.69	6.21	6.69	**6.69**	6.21	6.91	-	-	**0.0079***
Male	13.76	13.62	13.52	13.51	12.98	**13.52**	12.98	13.76	-	-	
**Age Group 50-54**	8.80	8.71	8.85	8.52	8.88	**8.80**	8.52	8.88	+0.2	0.8065	-
Female	6.21	6.50	6.51	5.82	6.07	**6.21**	5.82	6.51	-	-	**0.0079***
Male	11.92	11.37	11.49	11.55	11.95	**11.55**	11.37	11.95	-	-	
**Age Group 55-59**	7.68	7.72	7.79	7.20	7.49	**7.68**	7.20	7.79	-0.2	0.8065	-
Female	5.50	5.82	5.47	5.01	5.20	**5.47**	5.01	5.82	-	-	**0.0079***
Male	10.24	9.90	10.37	9.60	10.13	**10.13**	9.60	10.37	-	-	
**Age Group 60+**	7.01	6.90	7.10	5.73	5.76	**6.90**	5.73	7.10	-0.4	0.4624	
Female	5.56	5.41	5.97	4.37	4.28	**5.41**	4.28	5.97	-	-	**0.0079***
Male	8.68	8.54	8.27	7.22	7.48	**8.27**	7.22	8.68	-	-	
**Age Group 15-49**	10.29	10.11	10.04	9.53	9.53	**10.04**	9.53	10.29	-	-	-
Female	**7.63**	**7.35**	**7.18**	**6.72**	**6.69**	**7.18**	**6.69**	**7.63**	-	-	**-**
Male	15.22	14.37	14.39	13.51	12.98	**14.37**	12.98	15.22	-	-	-
**Province**											
Eastern Cape	8.95	8.90	9.12	8.96	8.25	**8.95**	8.25	9.12	-0.2	0.8065	-
Free State	9.33	9.05	8.14	7.94	8.82	**8.82**	7.94	9.33	-0.6	0.2207	-
Gauteng	8.91	8.63	8.26	7.62	7.34	**8.26**	7.34	8.91	-1.0	**0.0275***	-
Kwazulu-Natal	10.65	9.97	9.42	8.71	8.28	**9.42**	8.28	10.65	-1.0	**0.0275***	-
Limpopo	19.25	16.26	19.52	11.93	9.50	**16.26**	9.50	19.52	-0.6	0.2207	-
Mpumalanga	15.41	14.93	13.56	12.24	12.37	**13.56**	12.24	15.41	-0.8	0.0864	-
North West	10.82	9.51	8.79	8.95	8.86	**8.95**	8.79	10.82	-0.6	0.2207	-
Northern Cape	6.39	6.91	6.92	7.20	6.42	**6.91**	6.39	7.20	+0.4	0.4624	-
Western Cape	7.48	8.00	7.82	7.67	7.37	**7.67**	7.37	8.00	-0.4	0.4624	-

[a] Kendall's tau and p-values of correlation analyses in trends of HBsAg test positivity rates over time

[b] Mann-Whitney p-values generated from comparison of HBsAg test positivity rates between females and males by age group

*P-values < 0.05 in bold
