# Supplementary material for: Prevalence and incidence rates of laboratory-confirmed hepatitis B infection in South Africa, 2015 to 2019
Source: BMC Public Health. 2022 Jan 6;22:29. doi: 10.1186/s12889-021-12391-3 (PMC8739689; doi:10.1186/s12889-021-12391-3)
Supplement: Supplementary file 4 — Additional file 4. [file 12889_2021_12391_MOESM4_ESM.pdf]

Supplementary Table 4: Incidence of acute HBV and anti-HBc IgM testing rates, 2015 to 2019

Testing Year	Incidence (anti-HBc IgM positive cases) per 100,000 population								Anti-HBc IgM testing rate per 100,000 population							
	2015	2016	2017	2018	2019	2015 - 2019			2015	2016	2017	2018	2019	2015 - 2019		
						Median	Lower 95%CI	Upper 95% CI						Median	Lower 95%CI	Upper 955 CI
**Annual**	3.17	3.16	2.61	1.89	1.69	**2.61**	1.69	3.17	165.02	200.94	194.07	128.69	100.48	**165.02**	100.48	200.94
**Female**	3.26	3.35	2.61	1.88	1.62	**2.61**	1.62	3.35	186.84	226.84	218.29	136.67	103.36	**186.84**	103.36	226.84
**Male**	2.96	2.86	2.52	1.86	1.70	**2.52**	1.70	2.96	137.19	168.42	163.53	116.68	93.71	**137.19**	93.71	168.42
**Age Group 0-4**	0.03	0.05	0.03	0.05	0.07	**0.05**	0.03	0.07	23.64	20.91	22.57	19.39	18.49	**20.91**	18.49	23.64
Female	0.00	0.04	0.04	0.11	0.04	**0.04**	0.00	0.11	23.41	21.28	22.26	18.32	16.92	**21.28**	16.92	23.41
Male	0.07	0.07	0.03	0.00	0.10	**0.07**	0.00	0.10	22.63	19.93	22.05	19.36	19.39	**19.93**	19.36	22.63
**Age Group 5-9**	0.05	0.04	0.05	0.02	0.00	**0.04**	0.00	0.05	15.61	15.03	13.95	9.48	8.99	**13.95**	8.99	15.61
Female	0.07	0.04	0.04	0.04	0.00	**0.04**	0.00	0.07	16.73	16.72	14.92	9.91	9.40	**14.92**	9.40	16.73
Male	0.04	0.04	0.07	0.00	0.00	**0.04**	0.00	0.07	13.67	12.81	12.55	8.75	8.35	**12.55**	8.35	13.67
**Age Group 10-14**	0.06	0.06	0.04	0.04	0.04	**0.04**	0.04	0.06	23.90	23.58	23.25	15.85	12.42	**23.25**	12.42	23.90
Female	0.00	0.04	0.08	0.08	0.00	**0.04**	0.00	0.08	29.84	29.48	27.61	19.31	14.46	**27.61**	14.46	29.84
Male	0.13	0.08	0.00	0.00	0.07	**0.07**	0.00	0.13	16.83	17.25	18.46	12.08	10.11	**16.83**	10.11	18.46
**Age Group 15-19**	1.12	1.16	1.06	0.74	0.41	**1.06**	0.41	1.16	62.34	72.65	69.47	43.27	33.61	**62.34**	33.61	72.65
Female	1.79	1.86	1.61	1.18	0.73	**1.61**	0.73	1.86	91.44	109.34	102.05	62.03	45.16	**91.44**	45.16	109.34
Male	0.46	0.47	0.47	0.30	0.09	**0.46**	0.09	0.47	32.06	34.50	35.54	23.82	20.73	**32.06**	20.73	35.54
**Age Group 20-24**	8.04	7.52	6.11	3.60	2.38	**6.11**	2.38	8.04	164.29	192.61	184.59	115.57	81.42	**164.29**	81.42	192.61
Female	10.04	9.11	7.38	4.47	3.08	**7.38**	3.08	10.04	246.25	294.01	282.67	168.20	113.46	**246.25**	113.46	294.01
Male	5.58	5.84	4.78	2.66	1.62	**4.78**	1.62	5.84	79.27	88.65	83.84	61.48	47.06	**79.27**	47.06	88.65
**Age Group 25-29**	8.56	8.93	7.17	5.02	5.61	**7.17**	5.02	8.93	239.02	301.96	294.43	187.39	133.78	**239.02**	133.78	301.96
Female	8.92	9.53	7.45	5.10	5.62	**7.45**	5.10	9.53	328.33	412.51	404.98	247.18	167.06	**328.33**	167.06	412.51
Male	7.89	8.07	6.58	4.88	5.52	**6.58**	4.88	8.07	147.03	187.98	180.78	125.43	97.05	**147.03**	97.05	187.98
**Age Group 30-34**	6.05	6.11	5.44	4.38	3.50	**5.44**	3.50	6.11	310.90	376.16	366.58	224.39	159.05	**310.90**	159.05	376.16
Female	6.07	5.91	4.90	4.36	3.36	**4.90**	3.36	6.07	381.33	455.10	449.50	260.70	175.56	**381.33**	175.56	455.10
Male	5.91	6.13	5.80	4.33	3.50	**5.80**	3.50	6.13	236.82	293.32	280.10	184.20	137.90	**236.82**	137.90	293.32
**Age Group 35-39**	3.87	3.97	3.78	3.14	2.54	**3.78**	2.54	3.97	332.82	409.80	391.15	249.28	176.87	**332.82**	176.87	409.80
Female	3.39	4.13	3.24	2.92	2.31	**3.24**	2.31	4.13	356.29	439.83	424.71	254.61	178.67	**356.29**	178.67	439.83
Male	4.31	3.81	4.31	3.31	2.63	**3.81**	2.63	4.31	301.61	372.56	349.94	238.25	170.47	**301.61**	170.47	372.56
**Age Group 40-44**	2.54	3.07	2.00	1.85	1.81	**2.00**	1.81	3.07	305.25	384.77	368.15	240.07	181.51	**305.25**	181.51	384.77
Female	2.18	3.34	1.64	1.45	1.48	**1.64**	1.45	3.34	300.33	377.64	353.83	213.66	165.88	**300.33**	165.88	377.64
Male	2.86	2.79	2.25	2.21	2.15	**2.25**	2.15	2.86	303.90	384.76	375.47	262.45	191.92	**303.90**	191.92	384.76
**Age Group 45-49**	1.93	2.25	2.13	1.39	1.28	**1.93**	1.28	2.25	266.86	331.64	318.08	216.39	169.65	**266.86**	169.65	331.64
Female	1.74	2.19	1.90	1.03	1.45	**1.74**	1.03	2.19	248.33	304.46	291.62	187.06	147.39	**248.33**	147.39	304.46
Male	2.07	2.32	2.30	1.71	1.03	**2.07**	1.03	2.32	284.18	355.60	342.17	244.09	188.76	**284.18**	188.76	355.60
**Age Group 50-54**	2.15	1.30	1.33	0.80	1.14	**1.30**	0.80	2.15	223.25	283.49	267.63	185.14	150.96	**223.25**	150.96	283.49
Female	1.93	1.46	0.94	0.57	0.78	**0.94**	0.57	1.93	207.48	266.77	245.53	162.93	130.73	**207.48**	130.73	266.77
Male	2.43	1.01	1.73	1.08	1.59	**1.59**	1.01	2.43	237.37	299.64	291.06	209.99	171.70	**237.37**	171.70	299.64
**Age Group 55-59**	1.29	1.36	1.04	1.07	1.00	**1.07**	1.00	1.36	181.18	235.89	226.12	164.64	139.38	**181.18**	139.38	235.89
Female	0.79	1.45	1.50	0.90	0.56	**0.90**	0.56	1.50	165.39	212.14	196.65	143.01	118.59	**165.39**	118.59	212.14
Male	1.95	1.24	0.44	1.29	1.59	**1.29**	0.44	1.95	198.62	262.74	260.65	190.29	162.79	**198.62**	162.79	262.74
**Age Group 60+**	0.60	0.70	0.72	0.52	0.51	**0.60**	0.51	0.72	118.78	139.84	137.15	111.21	107.61	**118.78**	107.61	139.84
Female	0.52	0.64	0.75	0.38	0.22	**0.52**	0.22	0.75	98.34	117.36	114.47	90.15	88.50	**98.34**	88.50	117.36
Male	0.71	0.69	0.62	0.75	0.92	**0.71**	0.62	0.92	147.19	171.08	169.42	141.53	134.88	**147.19**	134.88	171.08
**Province**																
Eastern Cape	2.58	2.82	2.85	1.67	1.06	**2.58**	1.06	2.85	329.25	387.56	296.39	70.61	24.36	**296.39**	24.36	387.56
Free State	3.81	4.00	2.93	1.81	2.18	**2.93**	1.81	4.00	272.58	343.70	321.79	247.79	136.00	**272.58**	136.00	343.70
Gauteng	3.13	3.83	3.18	2.72	2.13	**3.13**	2.13	3.83	238.65	331.46	345.94	252.08	182.14	**252.08**	182.14	345.94
Kwazulu-Natal	4.82	4.00	3.28	2.24	2.63	**3.28**	2.24	4.82	102.15	80.70	87.70	81.25	88.68	**87.70**	80.70	102.15
Limpopo	1.45	1.42	0.70	0.59	0.50	**0.70**	0.50	1.45	48.90	79.10	66.77	46.80	35.89	**48.90**	35.89	79.10
Mpumalanga	3.99	4.00	3.51	1.57	1.11	**3.51**	1.11	4.00	46.26	83.03	117.74	43.78	43.16	**46.26**	43.16	117.74
North West	1.78	2.14	1.44	1.16	1.37	**1.44**	1.16	2.14	114.16	129.02	122.23	104.73	124.13	**122.23**	104.73	129.02
Northern Cape	3.83	2.96	2.11	3.60	1.42	**2.96**	1.42	3.83	336.25	343.91	327.64	320.52	262.53	**327.64**	262.53	343.91
Western Cape	2.45	1.87	1.79	1.23	1.26	**1.79**	1.23	2.45	76.57	69.65	62.67	50.41	49.60	**62.67**	49.60	76.57
