# Supplementary material for: Prevalence and incidence rates of laboratory-confirmed hepatitis B infection in South Africa, 2015 to 2019
Source: BMC Public Health. 2022 Jan 6;22:29. doi: 10.1186/s12889-021-12391-3 (PMC8739689; doi:10.1186/s12889-021-12391-3)
Supplement: Supplementary file 5 — Additional file 5. [file 12889_2021_12391_MOESM5_ESM.pdf]

Supplementary Table 5: Annual HBCoreIgM test positivity rates by gender, age group and province, 2015 to 2019

Testing Year	2015 (%)	2016 (%)	2017 (%)	2018 (%)	2019 (%)	Median (%)	2015 - 2019 Lower 95% CI	Upper 95% CI	Kendall's tau[a]	Kendall's p-value[a]	Mann-Whitney p-value[b]
**Annual**	1.92	1.57	1.35	1.47	1.68	**1.57**	1.35	1.92	-0.2	0.8065	-
**Female**	1.74	1.48	1.20	1.38	1.57	**1.48**	1.20	1.74	-0.2	0.8065	0.0952
**Male**	2.16	1.70	1.54	1.59	1.82	**1.70**	1.54	2.16	-0.2	0.8065	
**Age Group 0-4**	0.15	0.25	0.15	0.27	0.38	**0.25**	0.15	0.38	+0.8	0.0864	-
Female	0.00	0.17	0.16	0.58	0.21	**0.17**	0.00	0.58	-	-	0.8333
Male	0.30	0.35	0.16	0.00	0.53	**0.30**	0.00	0.53	-	-	
**Age Group 5-9**	0.35	0.24	0.38	0.18	0.00	**0.24**	0.00	0.38	-0.6	0.2207	
Female	0.43	0.21	0.24	0.36	0.00	**0.24**	0.00	0.43	-	-	>0.9999
Male	0.26	0.27	0.55	0.00	0.00	**0.26**	0.00	0.55	-	-	
**Age Group 10-14**	0.26	0.26	0.17	0.24	0.30	**0.26**	0.17	0.30	0.0	>0.9999	
Female	0.00	0.14	0.29	0.40	0.00	**0.14**	0.00	0.40	-	-	0.3819
Male	0.74	0.47	0.00	0.00	0.72	**0.47**	0.00	0.74	-	-	
**Age Group 15-19**	1.80	1.60	1.53	1.71	1.21	**1.60**	1.21	1.80	-0.6	0.2207	
Female	1.96	1.70	1.58	1.90	1.63	**1.70**	1.58	1.96	-	-	**0.0079***
Male	1.45	1.36	1.33	1.27	0.41	**1.33**	0.41	1.45	-	-	
**Age Group 20-24**	4.89	3.90	3.31	3.12	2.92	**3.31**	2.92	4.89	-1.0	**0.0275***	
Female	4.08	3.10	2.61	2.66	2.71	**2.71**	2.61	4.08	-	**-**	**0.0159***
Male	7.04	6.59	5.70	4.33	3.43	**5.70**	3.43	7.04	-	**-**	
**Age Group 25-29**	3.58	2.96	2.44	2.68	4.19	**2.96**	2.44	4.19	0.0	>0.9999	
Female	2.72	2.31	1.84	2.06	3.36	**2.31**	1.84	3.36	-	-	**0.0079***
Male	5.37	4.29	3.64	3.89	5.69	**4.29**	3.64	5.69	-	-	
**Age Group 30-34**	1.95	1.62	1.48	1.95	2.20	**1.95**	1.48	2.20	0.4	0.4624	
Female	1.59	1.30	1.09	1.67	1.91	**1.59**	1.09	1.91	-	-	**0.0079***
Male	2.50	2.09	2.07	2.35	2.54	**2.35**	2.07	2.54	-	-	
**Age Group 35-39**	1.16	0.97	0.97	1.26	1.43	**1.16**	0.97	1.43	0.4	0.4624	
Female	0.95	0.94	0.76	1.15	1.29	**0.95**	0.76	1.29	-	-	0.0556
Male	1.43	1.02	1.23	1.39	1.54	**1.39**	1.02	1.54	-	-	
**Age Group 40-44**	0.83	0.80	0.54	0.77	1.00	**0.80**	0.54	1.00	0.0	>0.9999	
Female	0.73	0.88	0.46	0.68	0.89	**0.73**	0.46	0.89	-	-	0.5476
Male	0.94	0.72	0.60	0.84	1.12	**0.84**	0.60	1.12	-	-	
**Age Group 45-49**	0.72	0.68	0.67	0.64	0.75	**0.68**	0.64	0.75	-0.2	0.8065	
Female	0.70	0.72	0.65	0.55	0.98	**0.70**	0.55	0.98	-	-	0.7381
Male	0.73	0.65	0.67	0.70	0.55	**0.67**	0.55	0.73	-	-	
**Age Group 50-54**	0.96	0.46	0.50	0.43	0.76	**0.50**	0.43	0.96	-0.2	0.8065	
Female	0.93	0.55	0.38	0.35	0.60	**0.55**	0.35	0.93	-	-	0.7302
Male	1.03	0.34	0.59	0.51	0.93	**0.59**	0.34	1.03	-	-	
**Age Group 55-59**	0.71	0.58	0.46	0.65	0.72	**0.65**	0.46	0.72	0.2	0.8065	
Female	0.47	0.68	0.76	0.63	0.47	**0.63**	0.47	0.76	-	-	0.7381
Male	0.98	0.47	0.17	0.68	0.97	**0.68**	0.17	0.98	-	-	
**Age Group 60+**	0.50	0.50	0.53	0.47	0.47	**0.50**	0.47	0.53	-0.4	0.4624	
Female	0.53	0.55	0.66	0.42	0.24	**0.53**	0.24	0.66	-	-	0.8810
Male	0.48	0.40	0.36	0.53	0.69	**0.48**	0.36	0.69	-	-	
**Province**											
Eastern Cape	0.79	0.73	0.96	2.37	4.34	**0.96**	0.73	4.34	+0.8	0.0864	-
Free State	1.40	1.16	0.91	0.73	1.60	**1.16**	0.73	1.60	-0.2	0.8065	-
Gauteng	1.31	1.16	0.92	1.08	1.17	**1.16**	0.92	1.31	-0.2	0.8065	-
Kwazulu-Natal	4.72	4.96	3.74	2.76	2.97	**3.74**	2.76	4.96	-0.6	0.2207	-
Limpopo	2.98	1.80	1.04	1.26	1.40	**1.40**	1.04	2.98	-0.4	0.4624	-
Mpumalanga	8.62	4.81	2.98	3.59	2.57	**3.59**	2.57	8.62	-0.8	0.0864	-
North West	1.56	1.66	1.18	1.11	1.10	**1.18**	1.10	1.66	-0.8	0.0864	-
Northern Cape	1.14	0.86	0.64	1.12	0.54	**0.86**	0.54	1.14	-0.6	0.2207	-
Western Cape	3.19	2.68	2.85	2.45	2.53	**2.68**	2.45	3.19	-0.1	0.2207	-

[a] Kendall's tau and p-values of correlation analyses in trends of HBCoreIgM test positivity rates over time

[b]Mann-Whitney p-values generated from comparison of HBCoreIgM test positivity rates between females and males by age group

*P-values < 0.05 in bold
